# A Novel Freshwater Cyanophage Mae-Yong1326-1 Infecting Bloom-Forming Cyanobacterium *Microcystis aeruginosa*

**DOI:** 10.3390/v14092051

**Published:** 2022-09-16

**Authors:** Fei Wang, Dengfeng Li, Ruqian Cai, Lingting Pan, Qin Zhou, Wencai Liu, Minhua Qian, Yigang Tong

**Affiliations:** 1 School of Marine Sciences, Ningbo University, Ningbo 315211, China; 2 College of Life Science and Technology, Beijing University of Chemical Technology, Beijing 100029, China

**Keywords:** *Microcystis aeruginosa*, cyanophage, genome, phylogenetic analysis

## Abstract

*Microcystis aeruginosa* is a major harmful cyanobacterium causing water bloom worldwide. Cyanophage has been proposed as a promising tool for cyanobacterial bloom. In this study, *M. aeruginosa* FACHB-1326 was used as an indicator host to isolate cyanophage from Lake Taihu. The isolated *Microcystis* cyanophage Mae-Yong1326-1 has an elliptical head of about 47 nm in diameter and a slender flexible tail of about 340 nm in length. Mae-Yong1326-1 could lyse cyanobacterial strains across three orders (*Chroococcales, Nostocales*, and *Oscillatoriales*) in the host range experiments. Mae-Yong1326-1 was stable in stability tests, maintaining high titers at 0–40 °C and at a wide pH range of 3–12. Mae-Yong 1326-1 has a burst size of 329 PFU/cell, which is much larger than the reported *Microcystis* cyanophages so far. The complete genome of Mae-Yong1326-1 is a double-stranded DNA of 48, 822 bp, with a G + C content of 71.80% and long direct terminal repeats (DTR) of 366 bp, containing 57 predicted ORFs. No Mae-Yong1326-1 ORF was found to be associated with virulence factor or antibiotic resistance. PASC scanning illustrated that the highest nucleotide sequence similarity between Mae-Yong1326-1 and all known phages in databases was only 17.75%, less than 70% (the threshold to define a genus), which indicates that Mae-Yong1326-1 belongs to an unknown new genus. In the proteomic tree based on genome-wide sequence similarities, Mae-Yong1326-1 distantly clusters with three unclassified *Microcystis* cyanophages (MinS1, Mwe-Yong1112-1, and Mwes-Yong2). These four *Microcystis* cyanophages form a monophyletic clade, which separates at a node from the other clade formed by two independent families (*Zierdtviridae* and *Orlajensenviridae*) of *Caudoviricetes* class. We propose to establish a new family to harbor the *Microcystis* cyanophages Mae-Yong1326-1, MinS1, Mwe-Yong1112-1, and Mwes-Yong2. This study enriched the understanding of freshwater cyanophages.

## 1. Introduction

Cyanobacterial bloom is a disastrous ecological phenomenon in which plankton, especially cyanobacteria, proliferate abnormally and gather on the water surface, causing water discoloration [1]. Due to anthropogenic activities, global warming, and eutrophication, cyanobacteria harmful algal blooms (cyanoHABs) are becoming increasingly extensive and frequent. Cyanobacterial blooms have many negative effects. Cyanobacteria blooms reduce the water surface clarity and thus inhibit the growth of aquatic macrophytes; cyanobacterial blooms reduce the dissolved oxygen content of water, resulting in the death of aquatic organisms, including fish, crab, shrimp, etc. [2]. Furthermore, cyanobacterial blooms make water toxic, as many cyanobacteria produce highly toxic secondary metabolites known as “cyanotoxin”. Cyanotoxins not only can notoriously cause liver and nervous system damage but also are immunotoxic, teratogenic, carcinogenic, and mutagenic [3,4,5,6,7,8,9]. Humans and animals can be exposed to cyanotoxins in various ways, such as through food, drink, inhalation, and dermal exposure during recreational activities. Therefore, it is urgent to solve the environmental problems caused by cyanobacterial bloom.

Cyanophages are phages that infect cyanobacteria. Phages are considered the most abundant biological entities on the planet, and their population is estimated to be 10^30^ to 10^32^ [10]. Cyanophage has been proposed as a promising tool for cyanobacterial bloom. The isolation and genomic analysis are the important basis for the research and application of cyanophages. In the past, studies on the isolation and genome analysis mainly focused on marine cyanophages, especially *Synechococcus* and *Prochlorococcus* cyanophages [11]. The research on freshwater cyanophages lags far behind. Little information about freshwater cyanophage can be found. Although nearly 350 cyanophage genomes have been reported, only 21reported cyanophages were isolated from freshwater. Among them, only 10 freshwater *Microcystis* cyanophages were reported. Only nine *Microcystis* cyanophage genomes (MaMV-DC, Ma-LMM01, Mic1, vB_MelS-Me-ZS1, PhiMa05, Mae-Yong924-1, MinS1, vB_MweS-yong2, and Mwe-Yong1112-1) have been sequenced and characterized [12,13,14,15,16,17,18,19,20]. Among them, five (MaMV-DC, Ma-LMM01, Mic1, Mae-Yong924-1, and MinS1) were isolated with *Microcystisaeruginosa. M. aeruginosa* is a major harmful cyanobacterium causing water bloom worldwide. It is very important to study virulent *M. aeruginosa* cyanophages.

In this study, *M. aeruginosa* FACHB-1326 was used as an indicator host to isolate cyanophage from Lake Taihu. The general features (morphology, one-step growth curve, physicochemical stabilities, and host range) of the isolated *Microcystis* cyanophage Mae-Yong1326-1 were analyzed. The complete genome of the isolated *Microcystis* cyanophage Mae-Yong1326-1 was sequenced and analyzed.

## 2. Materials and Methods

### 2.1. Isolation and Purification of Cyanophage

Cyanophage isolation was carried out according to the reported method [15]. The surface water samples were collected from Lake Taihu (North latitude, 31.246,376; East longitude, 120.371,044), Suzhou, China on 1 July 2021. The water samples were centrifuged at 10,000× *g* for 20 min at 4 °C. The supernatant was successively filtered through 0.45 µm and 0.22 µm pore size nitrocellulose membrane. Each 80 mL filtrate was mixed with 20 mL of 5 × BG11 liquid medium and 20 mL logarithmic-phase *M. aeruginosa* FACHB-1326 (OD680 ≈ 0.738, 2.34 × 10^7^ CFU/mL). In the control group, sterile water was substitute for the filtrate of water sample. The mixtures were cultured in a light incubator under a light/dark cycle of 12 h:12 h with a constant illumination of 30–40 µmol-photons/(m^2^ × s) at 25 °C until yellowing (about seven days). Lysates were centrifuged at 10,000× *g* for 10 min, and the supernatant was cultured again with fresh FACHB-1326 (about 2 × 10^7^ CFU/mL) until yellowing. Lysates were centrifuged at 10,000× *g* for 10 min. The supernatants were successively filtered through 0.45 µm and 0.22 µm pore size nitrocellulose filters. The filtrates were diluted (10^−1^–10^−9^) with BG11. Each 100 µL of dilution was mixed with 900 µL of logarithmic-phase FACHB-1326 cultures and incubated at 25 °C for 30 min, then mixed quickly with 8 mL of molten BG11 agar medium (0.7% agar, pre-incubated at 42 °C), and poured into a BG11 agar plate (1.5% agar). Clear plaques emerged in 7–10 days. Unique plaque was suspended in 3 mL of logarithmic-phase FACHB-1326 cultures and subsequently used for a new round of plaque isolation. Five rounds were carried out until plaques show uniform shape and size.

### 2.2. Transmission Electron Microscopy (TEM)

The cyanophage lysates were centrifuged at 10,000× *g* for 10 min. The supernatants were centrifuged at 35,000× *g* for 60 min. The precipitates were cleaned twice with 0.01 M PBS, suspended in PBS, and then deposited on a carbon-coated copper grid for 5 min, negatively stained with 3% uranyl acetate for 25 s, and observed under TEM (Hitachi-7650, Japan) as described [21].

### 2.3. One-Step Growth Curve Experiment

Fresh logarithmic-phase FACHB-1326 cultures (2.35 × 10^7^ CFU/mL) were mixed with Mae-Yong1326-1 suspension at optimal MOI of 0.1 in triplicates. After incubation for 30 min at 25 °C, the mixtures were centrifuged at 10,000× *g* for 10 min at 4 °C. The sediments were washed twice with BG11 and resuspended in an equal volume of BG11 medium. Samples were taken at 0, 30, 60, 120, 180, 360, 540, 720, 1440, 2160, and 2880 min, respectively. The titers in the samples were immediately determined using the double-layer plate method. The burst size of the *Microcystis* cyanophage Mae-Yong1326-1 was calculated as the ratio of the final number of released virions to the initial count of infected bacterial cells at the beginning of the latent period.

### 2.4. Physical and Chemical Tolerance Test

Temperature, pH, UV, and chloroform sensitivity assessment were performed. Aliquots of cyanophage stock solution (2.8 × 10^5^ PFU/mL) were adjusted to different pH (2, 3, 4, 5, 6, 7, 8, 9, 10, 11, 12) with NaOH or HCl, in triplicates and incubated for 2 h at 25 °C; aliquots of cyanophage stock solution (2.8 × 10^5^ PFU/mL) were incubated at 0 °C, 25 °C, 40 °C, 60 °C, and 80 °C, respectively, in triplicates. Samples were collected at 0 min, 20 min, 40 min, 60 min, 80 min, 100 min, and 120 min, respectively; aliquots of cyanophage stock solutions were irradiated under UV lamp (253.7 nm) in triplicates. Samples were collected at 0 min, 10 min, 20 min, 30 min, 40 min, 50 min, 60 min, 70 min, and 80 min respectively; aliquots of cyanophage stock solution (2.8 × 10^5^ PFU/mL) were added with chloroform at final concentrations (*v*/*v*) of 0%, 1%, and 2.5%, respectively, in triplicates. Control groups were added with an equal volume of 0.01 M PBS instead of chloroform. The mixtures were shaken and incubated in a light incubator for 30 min. Titers of the treated and untreated samples were measured using the double-layer plate method.

### 2.5. Host Range Experiments of Cyanophage

Thirty-nine freshwater cyanobacteria strains (Table 1) obtained from the Freshwater Algal Culture Bank of Institute of Hydrology (Wuhan, China), Academy of Sciences were used to determine the host range of cyanophage. In the experimental groups, each 300 μL of Mae-Yong1326-1 suspension (2.8 × 10^5^ PFU/mL) and 600 μL of cyanobacterial cultures in logarithmic growth phase were added to48-well plates in triplicates and incubated in the light incubator (25 °C, 2000 Lux, with a 12 h:12 h light–dark cycle). In the negative control group, the cyanophage suspension was replaced with BG11 medium. Three parallel experiments were performed. The lysis of the culture was observed daily, and OD_680_ measurements were also performed daily.

### 2.6. Genome Sequencing and Bioinformatics Analysis of the Cyanophage

The cyanophage lysate was centrifuged for 10 min at 10,000× *g*. The supernatant was filtered through a 0.22 μm nitrocellulose filter, pretreated with DNase (1 µg/mL) and RNase (1 µg/mL) for 2 h at 37 °C to remove host bacterial DNA and RNA, then incubated at 80 °C for 15 min. High Pure Viral kitA high Pure Viral kit (Roche, Product No: 11858882001) was used to extract the cyanophage genome. NEB Next Ultra II DNA Library PrepKit (NEB, Product No: E7645) for Illumina was used to construct a genomic library. Sequencing was performed using Illumina MiSeqsequencer (SanDiego, CA, USA) to obtain 2 × 300 bp paired-end reads. Trimmomatic V0.36 software was used to sift away low-quality sequencing reads (*Q* value < 20). De novo assembling was performed using SPAdes version V3.14.1 (http://cab.spbu.ru/software/spades/ (accessed on 16 August 2021)). Genome termini were analyzed as described previously [22] and using PhageTerm online (https://sourceforge.net/projects/phageterm (accessed on16 August 2021)) [23].

Mae-Yong1326-1 genome was annotated preliminarily with RAST (http://rast.nmpdr.org (accessed on 17 August 2021) [24]. All the predicted ORFs were verified manually by searching against the nr database with BLASTp (*E*-value < 10^−5^), searching against all the databases on the HMMER web server with hmmscan (https://www.ebi.ac.uk/Tools/hmmer/search/hmmscan (accessed on 17 August 2021)) [25] (benchmark: complete functional domain and *E*-value < 10^−5^) and searching against all the databases on the HHpred web server (https://toolkit.tuebingen.mpg.de/tools/hhpred (accessed on 5 April 202)) (benchmark: possibility > 96% and *E*-value ≤ 10^−5^) [26]. The tRNAscan-SE program was used to search for regions encoding tRNAs (http://lowelab.ucsc.edu/tRNAscan-SE/ (accessed on 5 April 2022)) [27]. Antibiotic resistance and virulence factor genes in Mae-Yong1326-1 genome were predicted in the CARD database (http://arpcard.mcmaster.ca (accessed on 5 April 2022)) and VFDB database (http://www.mgc.ac.Cn/VFs/main.htm (accessed on 5 April 2022)), respectively.

BLASTn alignment against nr database was used to searching sequences similar with Mae-Yong1326-1 genome. The pair-wise average nucleotide identity (ANI) values were calculated using OrthoANI (http://www.ezbiocloud.net/sw/oat (accessed on 8 April 2022)) [28]. To estimate the nucleotide sequence similarity between Mae-Yong1326-1 and other phages in current (5 January 2022) public databases, the Pairwise Sequence Comparison (PASC) classification tool (http://www.ncbi.nlm.nih.gov/sutils/pasc/ (accessed on 5 January 2022)) was used [29]. Nucleotide-based intergenomic similarities between Mae-Yong1326-1 and other phages in current (5 June 2022) public databases were also estimated by using VIRIDIC (http://rhea.icbm.uni-oldenburg.de/VIRIDIC/ (accessed on 5 June 2022)) [30]. Online software ViPTree (https://www.genome.jp/viptree/ (accessed on 8 April 2022)) [31] was utilized to generate a proteomic tree based on genome-wide similarities determined by tBLASTx.

## 3. Results

### 3.1. Isolation and Morphology of Cyanophage Mae-Yong1326-1

**Figure 1:**
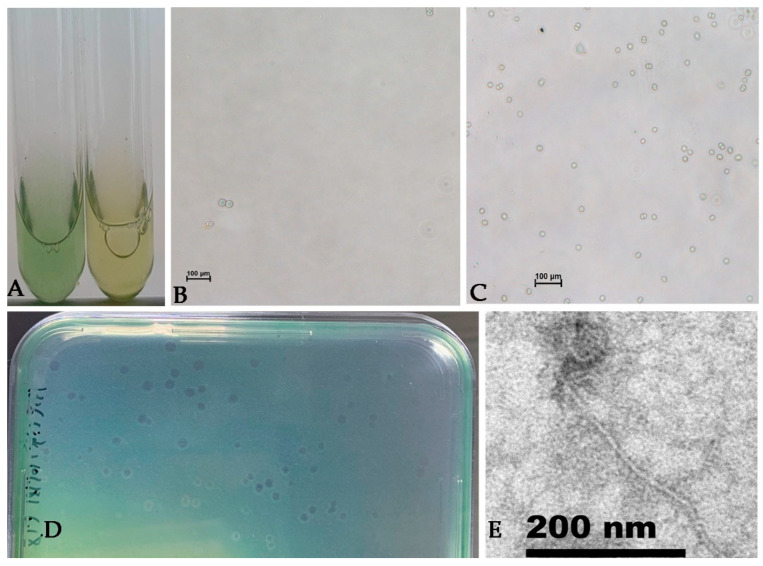
Micro- and macrographs of *M. aeruginosa* FACHB-1326 cultures, plaques, and negatively stained Mae-Yong1326-1. (**A**) Macrograph of a normal culture (left picture) and a *M. aeruginosa* FACHB-1326 culture infected with Mae-Yong1326-1 (right picture); (**B**) micrograph of a *M. aeruginosa* FACHB-1326 culture infected with cyanophage Mae-Yong1326-1. Scale bar =  100 µm; (**C**) micrograph of a normal culture of *M. aeruginosa* FACHB-1326. Scale bar =  100 µm; (**D**) plaques developed by Mae-Yong1326-1 on *M. aeruginosa* FACHB-1326 lawn; (**E**) a transmission electron micrograph of cyanophage Mae-Yong1326-1. Scale bar represents 200 nm.

The experimental group turned yellow in seven days (Figure 1A). The quantity of the cyanobacterial cells in the yellowing experimental groups (Figure 1B) was much less than that in the control group (Figure 1C) under microscopic observation. Mae-Yong1326-1 developed clear and circular plaques with diameter up to 5 mm in five days (Figure 1D). Cyanophage Mae-Yong1326-1 has an elliptical head of about 47 nm in diameter and a slender flexible tail of about 340 nm in length (Figure 1E).

### 3.2. One-Step Growth Curve

**Figure 2:**
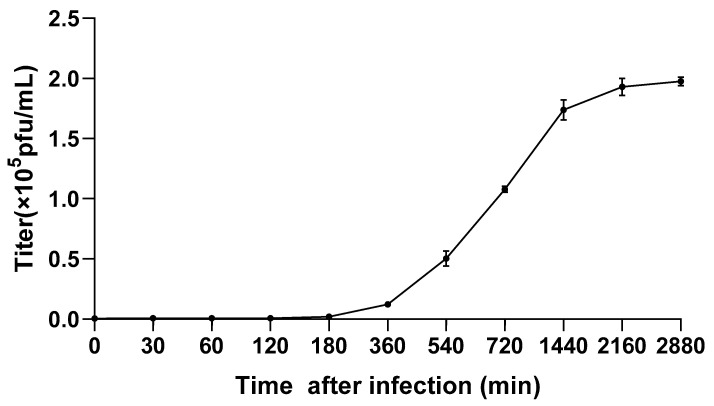
One-step growth curve of Mae-Yong1326-1 developed under the MOI of 0.1. Each dot represents the average titer at each time from the three parallel experiments. Error bars indicate standard deviations.

The one-step growth curve (Figure 2) of the cyanophage Mae-Yong1326-1 (at MOI = 0.1) showed that the titer of Mae-Yong1326-1 did not change significantly within 180 min post infection, increased slowly from 180 to 540 min, increased sharply from 540 to 1440 min, and remained relatively stable after 2160 min. Results indicated a latent period of 180 min and a burst period of 1980 min with the burst size of 329 PFU/cell [32].

The literature review revealed that the burst size of the previously reported *Microcystis* cyanophages ranged from 28-127 PFU/cell [17,18,33,34,35]. The burst size of Mae-Yong 1326-1 is much larger than them. That is, among all the *Microcystis* cyanophages studied so far, Mae-Yong 1326-1 has the largest burst.

### 3.3. Temperature, pH, UV and Chloroform Stability

**Figure 3:**
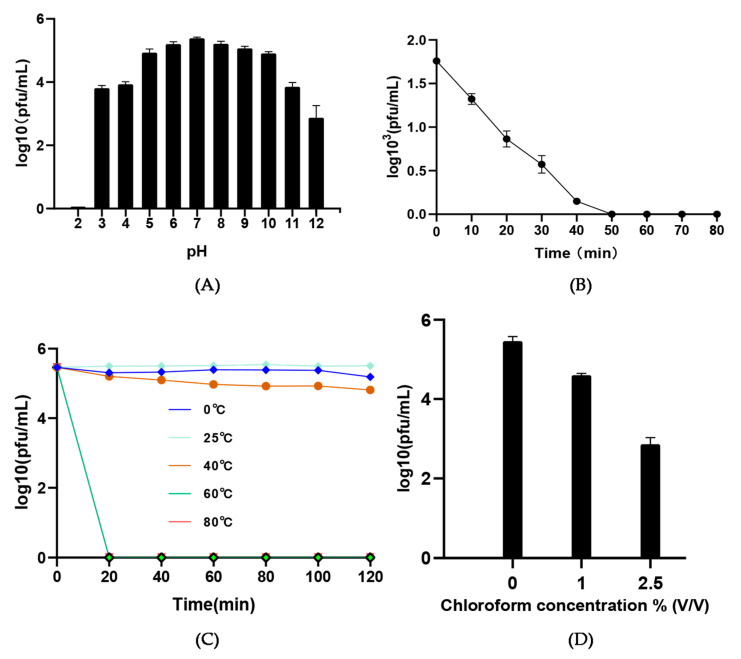
Physical and chemical tolerance test. (**A**) pH stability; (**B**) UV stability; (**C**) thermostability; (**D**) chloroform stability. All tests are performed in triplicate. Error bars indicate standard deviations.

The physicochemical stabilities (pH, UV, temperature, and chloroform) of cyanophages are important factors affecting the application potential. Mae-Yong1326-1 has a wide pH tolerance range. Its activity was relatively stable at pH3 to 12 although almost inactivated at pH 2 (Figure 3A). UV irradiation reduced the activity of Mae-Yong1326-1 and caused complete inactive in 50 min (Figure 3B). The activity of Mae-Yong1326-1 stayed at high levels at temperatures ranging from 0 °C to 40 °C, yet decreased to 0 within 20 min at the temperatures over 60 °C (Figure 3C). The best storage and transportation temperature for Mae-Yong1326-1 is room temperature (RT, 25 °C), as the activity of which was most stable at RT. Mae-Yong1326-1 maintained infectivity under chloroform treatment, but the activity of it decreased.

### 3.4. Host Range of Cyanophage Mae-Yong1326-1

The results of host range experiments showed that Mae-Yong1326-1 could lyse 7of the 39 tested cyanobacterial strains (Table 1). The susceptible cyanobacterial strains, across three taxonomic orders, were as follows: *M. aeruginosa* FACHB-1326, *M. aeruginosa* FACHB-924, and *M. wesenbergii* FACHB-908 of the order *Chroococcales*; *Aphanizomenon flos-aquae* FACHB-1209 and *Nostoc* sp. FACHB-596 of the order *Nostocales*; and *Planktothrix agardhii* FACHB-1261 and *Planktothricoides raciborskii* FACHB-881 of the order *Oscillatoriales*. Among the susceptible cyanobacteria, strains FACHB-1326, FACHB-924, FACHB-596, and FACHB-1261 were reported to be toxic [36,37,38].

Although most isolated cyanophages have a narrow host range, Mae-Yong1326-1 and four cyanophages, reported recently, have broad host range [15,16,18,20]. A wide host range may be advantageous for the application because cyanobacterial blooms are usually caused by multiple cyanobacteria [16].

### 3.5. General Characteristics of Mae-Yong1326-1 Genome

The average sequencing depth of Mae-Yong1326-1 genome was 616-fold. The complete genome of Mae-Yong1326-1 was a double-stranded DNA comprising 48,822 bp with 71.80% G + C content and long direct terminal repeats (DTR) of 366 bp. No tRNA gene was found in the genome. A total of 57 open reading frames (ORFs) in Mae-Yong1326-1 genome were predicted, with 31 on one strand and the other 26 on the opposite strand. All the ORFs covered 45,864 bp, resulting in a coding density of 93.99%. The average length of the coding products of the ORFs is 294 aminoacids (AA), with the smallest being 29 AA and the largest being 2441 AA (Table 2). Most ORFs (47 of 57, 84%) start with the initiation codon ATG, and the remaining 10 ORFs start with the initiation codon GTG. No known antibiotic-tolerance gene and virulence gene was found in Mae-Yong1326-1 genome, which proposes the security of the application potential of Mae-Yong1326-1 as a candidate for controlling *Microcystis* bloom. The genome was deposited in GenBank under the accession number OP028995.

**Figure 4:**
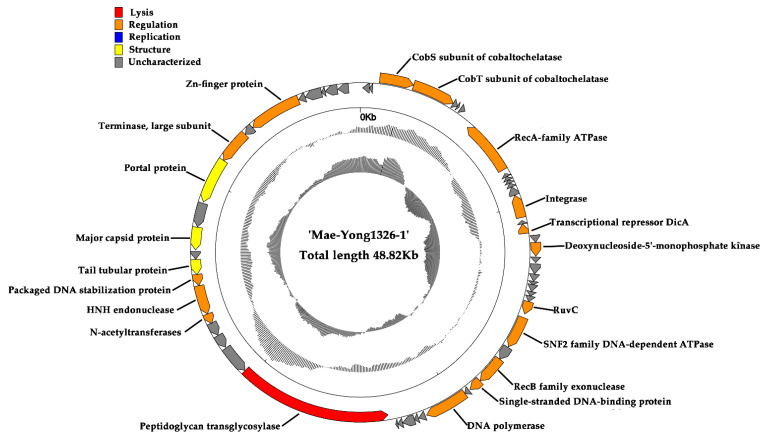
Genome map of *Microcystis* cyanophage Mae-Yong1326-1. The outermost circle represents 57 ORFs encoded in the genome, with different colors representing different functions (clockwise arrow indicates the forward reading frame; counterclockwise arrow indicates the reverse reading frame); the dark circles in the middle represent the GC content (Black indicates greater than the average GC content compared with the whole genome, and gray indicates the opposite); the innermost circle represents the GC skew (G − C/G + C: Outwards indicates > 0, and inwards indicates < 0).

By utilizing RAST, Blastp, HHpred, and HMMER, 20 ORFs in Mae-Yong1326-1 genome were predicted as known functional genes, accounting for about 35% of the total 57 ORFs. The remaining 37 ORFs, accounting for 65% of the total ORF, were unannotated. The annotated ORFs could be classified into four functional categories: DNA replication/regulation, structure, packaging, and lysis (Figure 4).

DNA replication and regulation genes: ORF 3 and ORF 4 of Mae-Yong1326-1 were predicted to encode CobS and CobT subunit of cobaltochelatase. *CobST* gene cluster is found to be widely encoded in tailed viruses that infect members of eight bacterial or archaeal orders [39]. In T4-like cyanophages, *cobST* gene cluster is part of the core genome [39,40,41], i.e., *cobS* and *cobT* genes are reported to be core genes in T4-likecyanophages, although *cobT* is usually mistakenly annotated as a peptidase [39,40].CobS and CobT were reported to play the role in the biosynthesis of cobalamin (vitamin B12), which is an important cofactor in various metabolic pathways, including DNA biosynthesis and replication of the virus [39,41]. ORF 52 encoded Zinc finger proteins that may be involved in transcriptional regulation or mediate protein–protein interactions [42]. ORF 44 encoded H-N-H endonuclease. HNH endonucleases were suggested to play an important role in the phage life cycle, fitness, and DNA packaging as well as in the response to environmental stress conditions [43].

Lysis, DNA packing, and structure genes: ORF 38 of Mae-Yong1326-1 was predicted to encode a putative peptidoglycan transglycosylase, which can crack the peptidoglycan cell wall of host cell [44]. ORF 50 encodes a putative terminase large subunit, which mediates DNA packaging and performs nuclease activity, thus generating the terminal of the phage chromosome [45]. ORF 43 was predicted to encode a packaged DNA stabilization protein, the function of which is involved with stabilizing the condensed DNA within the capsid [46]. ORF 45, 47, and 49 encoded putative tail tubular protein, major capsid protein, and portal protein, respectively.

### 3.6. Phylogenetic Analysis of Cyanophage Mae-Yong1326-1

**Figure 5:**
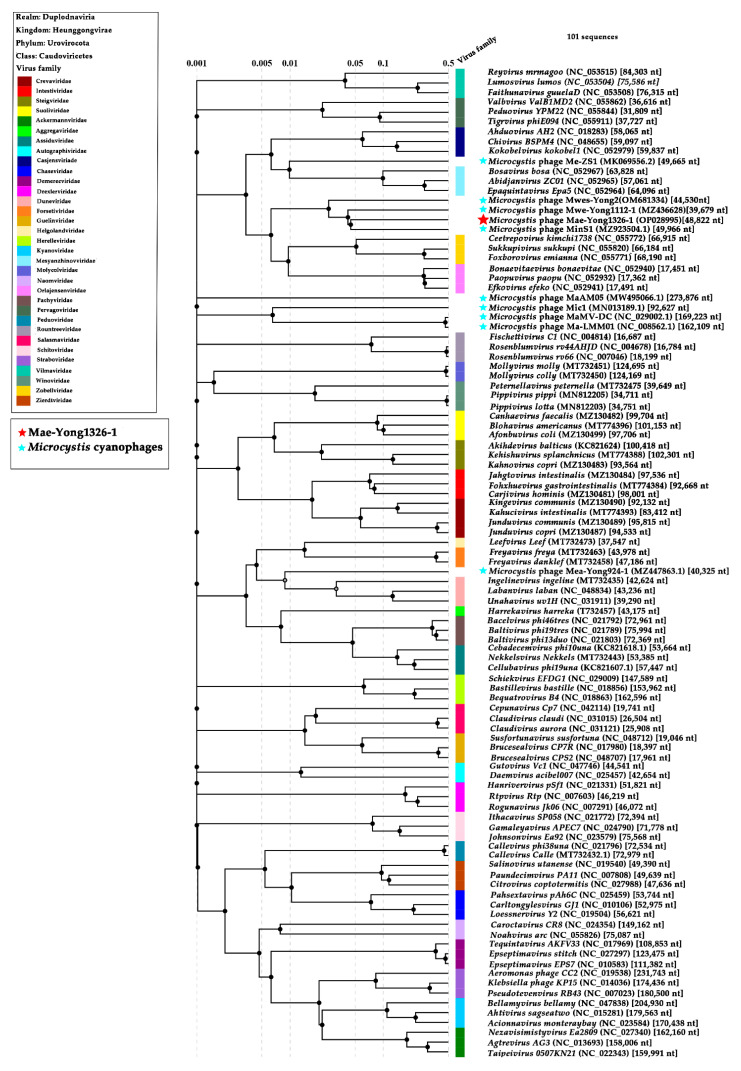
Phylogenetic proteomic tree of Mae-Yong1326-1, 9 reported *Microcystis* cyanophages, and 91 classified phages of the 33 families.

**Figure 6:**
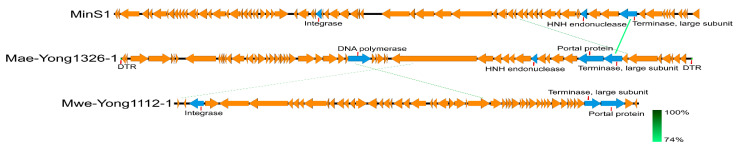
Genome comparison of the *Microcystis* cyanophage Mae-Yong1326-1, MinS1, and Mwe-Yong1112-1.

BLASTn search resulted that Mae-Yong1326-1 had the highest sequence similarity with *Leisingera* sp. NJS201 (accession number CP038234.1), but the query cover was close to 0; i.e., in practical terms, there is no homologous genome in the database. PASC is a web tool for the analysis of pairwise identity distribution within viruses [29]. PASC scanning with Mae-Yong1326-1 genome resulted that the maximum nucleotide sequence similarity between Mae-Yong1326-1 and the closest relative (*Microcystis* cyanophage MinS1) was only 17.75%, which was much lower than the threshold value of 70% to discriminate viral genus according to the International Committee on Taxonomy of Viruses (ICTV). In the VIRIDIC scanning with Mae-Yong1326-1 genome, the highest intergenomic similarities between Mae-Yong1326-1 and the closest relative (*Microcystis* cyanophage MinS1) was as low as 2%, which was far below the ≥70% boundary to define a genus. Results demonstrate that cyanophage Mae-Yong1326-1 reveals an unknown new genus. The ANI and isDDH values for *Microcystis* cyanophage Mae-Yong1326-1 and the closest relative, *Microcystis* phage MinS1, were only −1 and 12.5%, respectively. Terminase genes are considered to be a relatively conservative genes in *Caudoviricetes* class. In Blastp analysis, the large terminase subunit of Mae-Yong1326-1 shared only 55% of identity with the top hit and 33% with MinS1.The genome of a total of 91 classified phages of the class *Caudoviricetes* and the 9 reported freshwater *Microcystis* cyanophages were used as reference sequences to develop a proteomic tree, applying the online software ViPTree. In the proteomic tree (Figure 5) based on genome-wide sequence similarities, Mae-Yong1326-1 distantly clustered with three unclassified *Microcystis* cyanophages (MinS1, Mwe-Yong1112-1, and Mwes-Yong2). Like Mae-Yong1326-1, the highest similarities between MinS1, Mwe-Yong1112-1, Mwes-Yong2, and their closest relatives in PASC and VIRIDIC scanning were far below the ≥70% threshold to define a genus. Results indicated that *Microcystis* cyanophages Mae-Yong1326-1, MinS1, Mwe-Yong1112-1, and Mwes-Yong2 each reveal a new genus. These four *Microcystis* cyanophages form a monophyletic clade, separating at a node from the other clade formed by two families, *Zierdtviridae* and *Orlajensenviridae*, which are independent families of the *Caudoviricetes* class. Compared to Mwes-Yong2 and Mwe-Yong1112-1, the two *Microcystis* cyanophages, MinS1 and Mae-Yong1326-1, are more related. Genome comparison between Mae-Yong1326-1, MinS1, and Mwe-Yong1112-1 showed very low homology among them (Figure 6). Core Genes 5.0 (https://coregenes.ngrok.io/ (accessed on 10 July 2022)) analysis revealed no homologs shared by Mae-Yong1326-1, MinS1, Mwe-Yong1112-1, and Mwes-Yong2. Manual analysis revealed that all or some of them shared the homologs, including terminase, integrase, DNA polymerase, HNH endonuclease, and portal protein (Table 3). As mentioned earlier, *cobT* and *cobS* genes were reported to be core genes in cyanophages [39,40,41]. Yet, except Mae-Yong1326-1, no *cobT* or *cobS* genes were found in the genomes of Mwes-Yong2, MinS1, and Mwe-Yong1112-1 in bioinformatics analysis. In addition, unlike Mae-Yong1326-1, no fixed phage terminus and direct terminal repeat was found in Mwes-Yong2, MinS1, and Mwe-Yong1112-1 genomes. This corresponded with the very low nucleotide sequence similarity and intergenomic similarities among them. All the above results suggest more diverse characteristics of freshwater *Microcystis* cyanophages than have been previously known. We propose to establish a new family and four subfamilies to harbor the four *Microcystis* cyanophages including Mae-Yong1326-1, MinS1, Mwe-Yong1112-1, and Mwes-Yong2.

## 4. Conclusions

The newly isolated freshwater *M. aeruginosa* cyanophage Mae-Yong1326-1 is a novel virus species delegating a novel and genetically distinct evolutionary lineage of phages. This study enriches our understanding of freshwater cyanophage.

Mae-Yong1326-1 owns optimal characters beneficial to application. It is stable, maintaining high titers at 0–40 °C and at a wide pH range of 3–12. It has a big burst size of 329 PFU/cell, which is much larger than the reported *Microcystis* cyanophages. It has broad host range, capable of lysing toxic cyanobacterial strains across orders (*Chroococcales*, *Nostocales*, and *Oscillatoriales*). No Mae-Yong1326-1 ORF was found to be associated with virulence factor or antibiotic resistance.

## Figures and Tables

**Table 1:** Host range analysis of Mae-Yong1326-1 against 39 cyanobacteria strains.

Orders	Families	Species	Strains	Susceptible	Origin
*Chroococcales*	*Microcystaceae*	*Microcystis aeruginosa*	FACHB-905	−	China
			FACHB-942	−	China
			FACHB-469	−	France
			FACHB-924	+	Australia
			FACHB-925	−	Australia
			FACHB-1326	+	China
		*M. wesenbergii*	FACHB-908	+	China
			FACHB-929	−	Japan
			FACHB-1112	−	China
			FACHB-1318	−	China
			FACHB-1317	−	China
		*M. flos-aquae*	FACHB-1028	−	China
			FACHB-1351	−	China
			FACHB-1323	−	China
		*M. elabens*	FACHB-916	−	Japan
		*M. panniformis*	FACHB-1757	−	China
			FACHB-1409	−	China
		*M. viridis*	FACHB-979	−	Japan
			FACHB-1337	−	China
			FACHB-1342	−	China
		*Microcystis* sp.	FACHB-915	−	France
	*Chroococcaceae*	*Chroococcus* sp.	FACHB-193	−	China
*Nostocales*	*Aphanizomenonaceae*	*Aphanizomenon flos-aquae*	FACHB-1039	−	China
			FACHB-1209	+	China
			FACHB-1040	−	China
		*Dolichospermum flos-aquae*	FACHB-245	−	America
			FACHB-1255	−	China
			FACHB-418	−	France
	*Nostocaceae*	*Nostoc* sp.	FACHB-596	+	China
*Oscillatoriales*	*Microcoleaceae*	*Planktothrix agardhii*	FACHB-1166	−	China
			FACHB-920	−	Japan
			FACHB-1243	−	China
			FACHB-1261	+	China
	*Oscillatoriaceae*	*Oscillatoria planctonica*	FACHB-708	−	China
		*Planktothricoides raciborskii*	FACHB-881	+	China
*Synechococcales*	*Synechococcaceae*	*Synechococcus* sp.	PCC-7942	−	Australia
			FACHB-1061	−	China
*Hormogonales*	*Scytonemataceae*	*Plectonema boryanum*	FACHB-402	−	America
			FACHB-240	−	America

(+) representative infection; (−) representative non-infection.

**Table 2:** Functional prediction and top BLASTp hits of Mae-Yong1326-1 ORFs.

ORF	Size (aa)	Prediction Function	Top BLASTp Hit ^a^	Identity ^b^ (aa)	*E*-Values
1	106	Hypothetical protein	no hits		
2	49	Hypothetical protein	no hits		
3	508	CobS subunit of cobaltochelatase	gb|OJX48995.1|hypothetical protein BGO81_10395 [*Devosia* sp. 66–22]	58% (113/195)	1 × 10^−58^
4	644	CobT subunit of cobaltochelatase	gb|MAH25102.1|hypothetical protein [*Gammaproteobacteria* bacterium]	33% (38/114)	0.002
5	74	Hypothetical protein	no hits		
6	29	Hypothetical protein	gb|PSQ07931.1|beta-carotene 15,15′-dioxygenase [*Halobacteriales* archaeon QS56833]	80% (16/20)	0.18
7	78	Hypothetical protein	no hits		
8	878	RecA-family ATPase	ref|WP_171611044.1|AAA family ATPase [*Roseicella* sp. DB1501]	39% (112/286)	4 × 10^−42^
9	47	Hypothetical protein	no hits		
10	63	Hypothetical protein	ref|WP_032877434.1|hypothetical protein [*Pseudomonas* sp. BRG-100]	52% (32/61)	5 × 10^−13^
11	55	Hypothetical protein	no hits		
12	68	Hypothetical protein	ref|WP_184140002.1|DUF551 domain-containing protein [*Shinellafusca*]	62% (41/66)	1 × 10^−19^
13	139	Hypothetical protein	no hits		
14	367	Integrase	emb|CUW38828.1|putative Integrase (integrase-like, catalytic core,170–342) [*Magnetospirillum* sp. XM-1]	42% (143/338)	1 × 10^−67^
15	57	Hypothetical protein	no hits		
16	153	Transcriptional repressor DicA	tpg|HAO2892019.1|TPA: helix-turn-helix transcriptional regulator [*Escherichiacoli*]	70% (91/130)	2 × 10^−34^
17	107	Hypothetical protein	no hits		
18	203	Deoxynucleoside-5′-monophosphate kinase	seq gb|MCA6280837.1|deoxynucleotide monophosphate kinase [*Phenylobacterium* sp.]	48% (88/182)	7 × 10^−55^
19	69	Hypothetical protein	no hits		
20	41	Hypothetical protein	no hits		
21	51	Hypothetical protein	no hits		
22	107	Hypothetical protein	no hits		
23	149	Hypothetical protein	no hits		
24	87	Hypothetical protein	no hits		
25	88	Hypothetical protein	ref|WP_190872088.1|hypothetical protein [*Aulosira* sp. FACHB-615]	69% (60/87)	3 × 10^−36^
26	192	RuvC; Holliday junction resolvasomeRuvABC endonuclease subunit	gb|MBN9348280.1|DUF2815 family protein [*Devosia* sp.]	44% (68/156)	2 × 10^−29^
27	484	SNF2 family DNA-dependent ATPase	gb|MBF0421090.1|DEAD/DEAH box helicase [*Magnetococcales* bacterium]	44% (93/209)	4 × 10^−44^
28	649	Hypothetical protein	gb|MBN9348284.1|hypothetical protein [*Devosia* sp.]	46% (303/657)	3 × 10^−170^
29	425	RecB family exonuclease	gb|RPI18833.1|DUF2800 domain-containing protein [*Acidobacteriales* bacterium]	34% (129/375)	9 × 10^−44^
30	126	Single-stranded DNA-binding protein	gb|MBN9348280.1|DUF2815 family protein [*Devosia* sp.]	100% (126/126)	1 × 10^−87^
31	76	Hypothetical protein	no hits		
32	649	DNA polymerase	gb|MBN9348284.1|hypothetical protein [*Devosia* sp.]	46% (303/657)	3 × 10^−170^
33	100	Hypothetical protein	no hits		
34	61	Hypothetical protein	no hits		
35	180	Hypothetical protein	gb|EHM03436.1|hypothetical protein HMPREF9946_00111 [*Acetobacteraceae* bacterium AT-5844]	51% (76/148)	8 × 10^−35^
36	72	Hypothetical protein	emb|SKB62996.1|hypothetical protein SAMN06295937_1011120 [*Sphingopyxis flava*]	52% (37/71)	3 × 10^−13^
37	33	Hypothetical protein	no hits		
38	2441	Peptidoglycan transglycosylase	emb|CAB4120902.1|hypothetical protein UFOVP4_2 [uncultured Caudovirales phage]	34% (431/1278)	1 × 10^−174^
39	459	Hypothetical protein	no hits		
40	218	Hypothetical protein	no hits		
41	216	Hypothetical protein	gb|MBN9347258.1|hypothetical protein [*Devosia* sp.]	38% (58/151)	3 × 10^−24^
42	163	Acetyltransferase	gb|MBN9347259.1|hypothetical protein [*Devosia* sp.]	50% (78/157)	2 × 10^−41^
43	465	Packaged DNA stabilization protein	gb|MBN9347260.1|hypothetical protein [*Devosia* sp.]	39% (194/493)	2 × 10^−103^
44	176	HNH endonuclease	ref|WP_222211838.1|NUMOD4 domain-containing protein [*Burkholderiacepacia*]	49% (83/171)	1 × 10^−33^
45	228	Tail tubular protein	ref|WP_068432416.1|hypothetical protein [*Magnetospirillum* sp. XM-1]	44% (91/206)	3 × 10^−41^
46	727	Hypothetical protein	no hits		
47	358	Major capsid protein	gb|MBN9347263.1|phage major capsid protein	58% (212/366)	3 × 10^−140^
48	381	Hypothetical protein	gb|MBN9347264.1|hypothetical protein [*Devosia* sp.]	33% (84/251)	7 × 10^−21^
49	727	Portal protein	ref|WP_068432432.1|hypothetical protein [*Magnetospirillum* sp. XM-1]	48% (310/642)	0.0
50	532	Terminase, large subunit	ref|WP_068432438.1|phage terminase large subunit [*Magnetospirillum* sp. XM-1]	55% (281/510)	9 × 10^−175^
51	156	Hypothetical protein	no hits		
52	842	Zn-finger protein	ref|WP_237213204.1|hypothetical protein [*Roseomonas* sp. NPKOSM-4]	40% (155/386)	2 × 10^−47^
53	107	Hypothetical protein	ref|WP_174450698.1|hypothetical protein [*Azospirillumbaldaniorum*]	45% (49/110)	8 × 10^−18^
54	265	Hypothetical protein	gb|MBW8018009.1|hypothetical protein [*Planctomycetes* bacterium]	35% (41/118)	1 × 10^−5^
55	56	Hypothetical protein	no hits		
56	193	Hypothetical protein	no hits		
57	168	Hypothetical protein	gb|MBV9984493.1|hypothetical protein [*Bradyrhizobium* sp.]	36% (52/144)	8 × 10^−12^

^a^ the most closely related protein and its organism. “No hits” indicates no significant hits. ^b^ percent identity for the top hits in BLASTP scanning. Numbers in parentheses provide length of each alignment.

**Table 3:** Genome-characteristics of *Microcystis* cyanophages Mae-Yong1326-1, MinS1, Mwe-Yong1112-1, and Mwes-Yong2.

Cyanophage	Indicate Host	Size (bp)	G + C	Fixed Terminus	DTR	Terminase	Integrase	DNA Polymerase	HNH Endonuclease	Portal Protein
Mae-Yong1326-1	*M. aeruginosa*	48.822	71.8%	Yes	366 bp	Y	Y	Y	Y	Y
MinS1	*M. aeruginosa*	49.996	71.8%	No	no	Y	Y	N	Y	N
Mwe-Yong1112-1	*M. wesenbergii*	39.679	66.6%	No	no	Y	Y	N	N	Y
Mwes-Yong2	*M. wesenbergii*	44.530	71.6%	No	no	Y	Y	Y	Y	Y

(Y) indicates that there is/are ORF/ORFs annotated with this function in the genome; (N) indicates that no ORF was annotated with this function.
